# Effect of Post-Infiltration Soil Aeration at Different Growth Stages on Growth and Fruit Quality of Drip-Irrigated Potted Tomato Plants (*Solanum lycopersicum*)

**DOI:** 10.1371/journal.pone.0143322

**Published:** 2015-12-02

**Authors:** Yuan Li, Zongxia Jia, Wenquan Niu, Jingwei Wang, Mingzhi Zhang

**Affiliations:** 1 Institute of Soil and Water Conservation, Northwest A&F University, Yangling, 712100, Shaanxi, China; 2 Institute of Soil and Water Conservation, Chinese Academy of Sciences & Ministry of Water Resources, Yangling, 712100, Shaanxi, China; 3 College of Water Resources and Architectural Engineering, Northwest A&F University, Yangling, 712100, Shaanxi, China; 4 Institute of Water-saving Agriculture in Arid Areas of China, Northwest A&F University, Yangling, 712100, Shaanxi, China; 5 Dezhou Prospecting and Designing Institute of Water Conservancy, Dezhou, 253000, Shandong, China; Purdue University, UNITED STATES

## Abstract

Soil hydraulic principles suggest that post-infiltration hypoxic conditions would be induced in the plant root-zone for drip-irrigated tomato production in small pots filled with natural soil. No previous study specifically examined the response of tomato plants (*Solanum lycopersicum*) at different growth stages to low soil aeration under these conditions. A 2 × 6 factorial experiment was conducted to quantify effects of no post-infiltration soil aeration versus aeration during 5 different periods (namely 27–33, 34–57, 58–85, 86–99, and 27–99 days after sowing), on growth and fruit quality of potted single tomato plants that were sub-surface trickle-irrigated every 2 days at 2 levels. Soil was aerated by injecting 2.5 liters of air into each pot through the drip tubing immediately after irrigation. Results showed that post-infiltration aeration, especially during the fruit setting (34–57 DAS) and enlargement (58–85 DAS) growth stages, can positively influence the yield, root dry weight and activity, and the nutritional (soluble solids and vitamin C content), taste (titratable acidity), and market quality (shape and firmness) of the tomato fruits. Interactions between irrigation level and post-infiltration aeration on some of these fruit quality parameters indicated a need for further study on the dynamic interplay of air and water in the root zone of the plants under the conditions of this experiment.

## Introduction

It is well known that mesophytic plant roots access soil O_2_ (in air or dissolved) by diffusive mass transfer. At 298 °K the diffusion coefficient for O_2_ in water is 2.6 ×10^−5^ cm^2^ s^-1^ compared to 0.176 cm^2^ s^-1^ in air[1,2]. This implies that under the same O_2_ concentration gradient the diffusive mass transfer is close to 6800 times greater in air than in water.

Plant roots therefore depend primarily on the oxygen supply from soil air which can be continually replenished by exchange with the atmosphere, rather than from oxygen dissolved in the soil solution.

Tomato (*Solanum lycopersicum L*.*)* is cultivated in China under small (0.1 to 0.2 ha), low-cost, structures with removal plastic covers[3,4]. Container (or pot) production systems that use natural soil media are less expensive and common in China's principal tomato production provinces (namely Shandong, Xinjiang, Hebei, and Henan). Since water and air occupy the same pore space of the soil volume in the pots, their soil saturation levels are reciprocally related. Increasing water saturation implies decreasing air saturation and vice-versa. In this context, formulating irrigation timing, level, and frequency for potted plants needs to take into account meeting crop water and nutrient requirements as well as preventing oxygen-deficient conditions in the root environment. These choices would depend on many factors such as the irrigation method, irrigation water quality, soil type, and crop species.

Soil hydraulics dictate that gravitational drainage from pots can take place only if the soil layer at the bottom of the pots is saturated (Richard's outflow law) and the profile depth in the pot exceeds the soil bubbling pressure. As a result, the soil hydraulics in containers is different from natural soil profiles, and pot production systems are prone to varying periods of exposure to low oxygen levels (hypoxia) or lack of oxygen (anoxia) in the root zone following irrigation. Finer soil textures would pose a higher risk because of their higher bubbling pressure and slower post-infiltration internal drainage and redistribution than coarse sands. Small pots require less soil and occupy less space under covered production. On the other hand, they would provide less soil volume for root expansion and higher risk of exposure to hypoxia following irrigation. In this case, topping and/or complete or partial pruning of lateral shoots and trusses (especially for indeterminate cultivars) are used to limit shoot growth and root expansion and improve fruit size and quality[5,6].

Several studies in the literature support the ideas in the preceding discussion. Bhattarai et al. [7] presented an in-depth review of studies showing that irrigation with aerated or oxygenated water (termed as airgation and oxygation) or addition of hydrogen peroxide[8,9] would increase dissolved O_2_ content and offset the negative effects of hypoxia following irrigation. Wang et al.[10] demonstrated that partitioning the root system of maize plants in containers divided into two compartments and alternately irrigating them, lessened the possible effect of hypoxic or anoxic conditions compared to single compartment pots.

Subsurface drip (or trickle) irrigation of potted plants not only requires less water but keeps the soil surface partially saturated and open to diffusion of atmospheric air. Also, the saturated root-zone volume below the level of the emitters can be controlled by varying the irrigation rate and frequency[11]. Depending on species, roots of mesophytes can tolerate varying periods of hypoxia or anoxia and may even acclimatize and develop greater tolerance following exposure[12,13]. Sensitivity to low rhizosphere soil aeration for a given species may also change over time, and therefore plant response may also depend on different growth stages of the crop. Under hypoxic conditions in the root zone, above-ground symptoms may not be apparent although physiological changes have occurred that can decrease growth and development and lower yield. Root zone hypoxia causes stomatal closure[14], and this in turn results in less gas exchange with leaves, lower photosynthesis and transpiration along with reduced water and nutrient uptake. Other associated effects are reduced root mass and leaf expansion, and visible loss of leaf chlorophyll and premature leaf senescence[15,16,17,18].

Tomato plants (*Solanum lycopersicum*) are one of the most vulnerable mesophytes to hypoxia in the root environment[19]. High vulnerability incorporates the concepts of low sensitivity (i.e. low negative response thresholds) and low resilience (i.e. ability to adjust, recover, or resist a period of exposure). There are no reports in the literature on the inter-relationship of these two aspects of vulnerability of tomato plants to root zone hypoxia nor reports on plant characteristics and environmental factors that contribute to, or are associated with, high vulnerability. Rao and Li[20] reviewed studies on negative effects of root zone flooding on tomato plants. Sensitivity of the plants according to their growth stage were in the order flowering stage > fruiting setting and fruit growth stage > seedling stage > fruit ripening stage but this order depended on duration of exposure to hypoxic conditions. Negative responses included reduced plant height, leaf area, leaf chlorophyll content, fruit set, and respiration rate.

A literature search returned few studies on the effects of root-zone hypoxia on tomato fruit market quality and nutritional value[11,21,22]. These studies indicate aerated irrigation (termed airgation) can increase shoot growth along with yield, hardness, and nutritional value of tomato fruit. A similar paucity of studies exists on tomato root growth response to hypoxia. Aeration through subsurface trickle irrigation tubes stimulated root growth[23,24,25] and artificial aeration has been shown to promote root metabolism and growth[26,27]. Meek et al.[28] reported higher yields of tomatoes were related to higher oxygen contents in the soil root zone before and during the fruit-enlarging stage.

The foregoing discussion indicates that post-infiltration aeration may be beneficial for subsurface drip-irrigated potted tomato(*Solanum lycopersicum*) production using natural soil. Also, that this benefit may be realized with aeration restricted to specific growth stages. An ongoing research program at Northwest Agriculture and Forest University, Yanling, China addresses issues with drip-irrigated production of vegetables in containers [11,27]. To date, no study has specifically examined the sensitivity of tomato plants(*Solanum lycopersicum*) to low soil aeration at different growth stages and how this may impact root and fruit growth, development, and quality. However, such information would have high practical value, if it shown that post-infiltration aeration is needed only at specific growth stages of tomato plants to overcome the negative effects of hypoxia in the root zone under sub-surface trickle-irrigation.

This objective of this study was to quantify effects of no post-infiltration soil aeration versus aeration during 5 different periods (namely 27–33, 34–57, 58–85, 86–99, and 27–99 days after sowing) on potted tomato plants(*Solanum lycopersicum*) that were sub-surface trickle-irrigated every 2 days at 2 levels. Specifically, we examined data on yield, root growth and activity, and the market and nutritional value of the fruits.

## Materials and Methods

### Site description

The experiment was conducted under a rain-shelter at the Key Laboratory of Water and Soil Engineering for Dry Regions at Northwest A & F University Yangling, Shaanxi, China between April 7 and July 15, 2011. The location is semi-arid with an average annual rainfall of 572.5 mm, an average annual sunshine of 2163.8 hours and 210 frost-free days.

### Experimental design

On April 20, 3-week old tomato seedlings of the variety “*Tianze Chunlei*” were transplanted to 144 experimental pots. Each pot contained topsoil collected from the 0 cm to 20 cm depth of the Key Laboratory farmland (a Lou silty clay loam classified as Inceptisol based on the U.S. soil taxonomy). Three liters of water were added to each pot after transplanting. The pots had an upper inner diameter of 29 cm, a bottom inner diameter of 22 cm and a depth of 24.5 cm and were filled with soil to 22.5 cm to achieve a dry bulk density of 1.3 g cm^-3^. The total volume (V) of soil in the pots was 9.8 liter calculated as the volume of a truncated right circular cone of large diameter = 25.2 cm (R), small diameter 22 cm (r), and height 22.5 cm (h) using the formula V = [πh (R^2^ + r^2^ + Rr)]/3. The weight of soil in each pot was 12.75 kg. The field capacity, topsoil initial moisture, total nitrogen, and organic matter contents were 230 g kg ^-1^ (23% by weight), 23.8%, 0.98 g kg^-1^ and 9.51 g·kg^-1^, respectively. The total porosity (n) of the silty clay loam soil in the pots was estimated as 1 minus the ratio of the dry bulk density (ρ_b_) to the particle density (ρ_p_). Using the measured ρ_b_ = 1.3 g cm^-3^ and taking ρ_p_ = 2.60 g cm^-3^ gives an estimated n = 0.50 Every 2 days, starting April 27 (27 days after sowing), 72 pots were irrigated at 0.6 to 0.7 of the gravimetric field capacity (low level) and the other 72 pots at 0.7 to 0.8 of field capacity (high level). This implies the weight of water in the pots at the low irrigation level ranged from 1.76 (= 0.6 ×0.23 ×12.75 kg) to 2.05 kg (0.7 ×0.23 ×12.75 kg). Similarly, weight of water in the pots at the high irrigation level ranged from 2.05 kg to 2.35 kg. The volume of water needed for the 2 irrigation levels was approximated through weighing[29] and recorded for each pot. The five post-infiltration aeration periods by date and days after sowing (DAS) were: (1) the seedling stage between May 4 and 10 or 27 to 33 DAS, (2) the flowering and fruit setting stage between May 10 and June 3 or 34–57 DAS, (3) the fruit enlarging stage between June 3 and July 1 or 58–85 DAS, (4) the final fruit ripening stage between July 1 and 15 or 86–99 DAS, and (5) the whole growing period between May 4 and July 15 or 27 to 99 DAS. During these post-infiltration aeration periods, air was injected into the pots through the buried drip irrigation tubes with an air compressor at a rate of 2.5 liters per pot. This rate approximated 50% of the soil porosity in each pot. A no-aeration control was included for a total of 6 experimental treatments. For each irrigation level, each of the 6 treatments was applied to a block of 12 pots. The tomato plants(*Solanum lycopersicum*) were pruned to single stems, and were topped after the appearance of 4 trusses. Except irrigation and aeration, the other management measures were the same for all pots.

Three pots from each treatment block were tagged for ripe fruit yield, fruit characteristics, and other non-destructive measurements. The remaining 9 pots in each block were included for destructive measurements at end of the first three growth periods (namely 27–33, 34–57, and 58–85 DAS). At end 57 and 85 DAS, three pots were randomly sampled from each treatment block for destructive measurements of root dry weight and root vigor. The roots of the plants were removed and washed through a sieve. Root activity was measured on a 0.5-g portions of fresh root tips using the triphenyl tetrazolium chloride (TTC) method[30]. This test is based on live roots reducing colorless 2,3,5-triphenyl tetrazolium chloride (TTC) to red triphenyl formazan (TPF). The latter is then extracted after a fixed incubation period and measured. Root activity was expressed as the mass TPF produced in mg per g fresh root per hour. The dry weight of the washed roots was then measured. This was also done for the 3 tagged plants at 99 DAS after the final fruits were harvested.

The volume of water for the 38 irrigations applied to the 3 tagged pots for each treatment were totaled and averaged to obtain a mean value of water applied per pot (n = 18) for the low and high irrigation levels.

### Fruit quality measurements

Fully ripe fruits on the tagged plants were progressively harvested and weighed until 99 DAS. The size and hardness of the first 8 ripe fruits harvested from the first truss were determined. Axial and lateral fruit diameter of each fruit (mm) were measured using a Vernier caliper. Fruit hardness (N mm^-2^) was measured in the equatorial area of each fruit using a KM H-51 penetrometer (Kiya Seisakusho Co. Ltd, Tokyo, Japan).

The taste and nutritional properties of the first ripe fruit harvested from the first truss of each tagged plant were determined. The fruit was sliced and blended after removing the skin and seeds. A hand-held ATAGO-P32 temperature-compensated refractometer (ATAGO Co. Ltd, Tokyo, Japan) was used to directly read the % soluble solids (as °Brix) of the blended fruit at room temperature[31]. Titratable acids (% by weight) were determined by diluting an aliquot of the blended fruit and titrating against 0.1 M NaOH using phenolpthalein as an indicator. The % by weight titratable acids was estimated as (ml NaOH ×acid factor = 0.0064) divided by ml aliquot of blended fruit. Because skin and seeds make up less than 1% of tomato fruit fresh weight, these parameters measured in weight % of the blended fruit can be taken for practical purposes as % by weight of fresh fruit[3,32]. Vitamin C content (mg per 100 g fresh fruit) was determined by molybdenum blue colorimetry[33]. This method is based on the reaction of ascorbic acid (VC) with ammonium phospho-molybdate in the presence of SO_4_^2-^ and PO_4_^3-^ generating the blue molybdenum. This blue molybdenum has the maximum absorption at 760 nm. This vitamin C assay method is accurate, repeatable, and insensitive to the interference in the presence of common reducing sugars.

### Statistical analysis

The experimental design was treated as a 2 ×6 factorial with 3 replicates for statistical analyses. Accordingly, the data collected for each of the observed experimental variables was analyzed using the general linear model Y_ijk_ = μ + α_i_ + β_j_ + (αβ)_ij_ + ε_ijk_ where Y_ijk_ denotes the variable observed on the k^th^ replicate (k = 1, 2, 3) for the i^th^ irrigation (i = 1, 2) and j^th^ aeration treatment (j = 1, 2 … 6). Here μ denotes the overall mean of the variable, α_i_ the effect of irrigation, β_j_ the effect of aeration, (αβ)_ij_ the irrigation ×aeration interaction effect, and ε_ijk_ the associated random error. The fitting of the model was done using 2 way ANOVA routine of the SPSS software package (IBM, Armonk, New York). If the ANOVA results for a given response showed no significant irrigation ×post-infiltration aeration effect, the aeration treatment marginal means were separated by the Duncan multiple range test. A t-test was used to compare irrigation treatment marginal means. If significant interaction effects were obtained, the simple effect aeration treatment means of at each irrigation level were separated by the Duncan multiple range test.

## Results

### Fresh fruit yield, soluble solids and plant growth

About 5 to 6 medium-sized high-quality fruits were harvested from each pot as a result of the heavy pruning of the stems and culling of the trusses. Neither post-infiltration aeration nor irrigation level treatments significantly impacted (P ≤ 0.05) fresh fruit yield of the potted single tomato plants (Table 1). The ANOVA showed there was no irrigation ×post-infiltration aeration interaction effect. Nevertheless, fresh fruit yields for all the post-infiltration aerated plants were higher than those that received no aeration treatment. For the low irrigation treatment, the mean yield (n = 3) of the non-aerated plants was 616 g per plant compared to the mean yield for the of 688 g per plant (about 12% higher) for the pots that were aerated for the entire period of 27–99 days after sowing. Corresponding values for the high irrigation treatment were 633 and 693 g per plant (about 9% higher). The mean yield (n = 18) across all aeration treatments was 674 g per plant at the low irrigation level and 680 g per plant at the high irrigation level.

**Table 1:** Fruit yield, above-ground plant dry weight (g per plant), plant height (cm) and soluble solids content of the first ripe fruit from the first truss of potted single tomato plants for 6 post-infiltration aeration treatments (i.e. none or 2.5 liter aeration applied during 5 different periods) at 99 DAS. Pots were subsurface drip-irrigated at 2-day intervals to maintain the soil at 60 to 70% or 70 to 80% of volumetric field capacity (denoted as low and high irrigation level).

Aeration period, days after sowing	Fruit yield,(g per plant)			Plant dry weight,(g per plant)			Plant height,(cm)			Soluble Solids(°Brix)		
Irrigation level	Low	High	Mean(n = 6)	Low	High	Mean(n = 6)	Low	High	Mean(n = 6)	Low	High	Mean(n = 6)
None	616a	633a	625a	30.7b	36.0a	33.3c	78.2d	79.1c	78.6c	4.67a	4.73a	4.70a
27–33	680a	683a	681a	40.2ab	41.0a	40.6abc	80.5d	84.5b	82.5b	5.00ab	5.20abc	5.10abc
34–57	695a	704a	699a	42.2a	52.3a	47.3a	88.8ab	88.8a	88.8a	5.13ab	5.73bc	5.43cd
58–85	709a	710a	709a	44.1a	51.5a	47.8a	90.8a	88.0a	89.4a	5.47b	5.80c	5.63d
86–99	654a	659a	657a	36.1ab	37.9a	37.0bc	82.2cd	83.1b	82.7b	4.87ab	4.93ab	4.90ab
27–99	688a	693a	691a	40.5ab	49.2a	44.9ab	85.0bc	82.2b	83.6b	5.13ab	5.40abc	5.27bcd
OVERALLMEAN	674	680	677	39.0ab	44.6	41.8	84.2	84.3	84.3	5.04	5.30	5.17

*Notes*: Irrigation and interaction effects were not significant (p>0.05) in ANOVA. Aeration treatment means across irrigation levels (n = 6) not followed by the same letter are significantly different at the 5% level.

Even the short periods of post-infiltration aeration tended to increase fresh fruit yield. As shown in Table 1 aeration from 27 to 33 DAS resulted in a 10% yield increase from 616 to 680 g per plant for the low irrigation level. For the high irrigation level, there was a corresponding 8% increase from 633 to 683 g per plant. Lower increases of 6% and 4% over the no aeration treatment were obtained with aeration from 86 to 99 DAS (Table 1). Maximum yields of 709 and 710 g per plant were obtained for post-infiltration aeration during 58–85 days after sowing corresponding to the fruit setting period (Table 1).

In general, above ground plant dry weight and plant height at 99 DAS (Table 1) mirrored the yield response to the treatments. This would be expected since these three variables represent an integrated measure of the treatment effects on the plant over the entire growth period. The 2-way ANOVA showed no significant interaction between irrigation level and post-infiltration aeration treatments. Overall mean above ground plant dry weight for the high irrigation level was 14% higher than the lower level (Table 1). As was found for fresh fruit yield, post-infiltration aeration even for short periods resulted in above ground dry weight and plant height increases although these increases were statistically non-significant. Also, highest increases for plant dry weight and height over the no-aeration treatment occurred for the longer periods of post-infiltration aeration.

The 2-way ANOVA results (Table 1) indicated a highly significant effect (P = 0.004) of post-infiltration aeration effect on soluble solids content (as °Brix) of the fresh fruits. Irrigation level effects were borderline significant (p = 0.058), and there was no significant interaction between the two experimental treatment factors on °Brix. The LSD_0.05_ for comparing irrigation mean values (n = 18) for soluble solids content across all aeration treatments was 0.26. The overall means (Table 1) for the irrigation treatment on soluble solids content values were 5.04 versus 5.30 °Brix.

Soluble solids values at the high irrigation level were greater than those at the low level for all aeration treatments (Table 1). Soluble solids content responded positively to post-infiltration aeration during the flowering (34–57 DAS) and fruit setting stages (58–85 DAS). Values at the low irrigation level for these treatments were 10% and 17% higher than without aeration. Corresponding values were 21% and 23% at the high irrigation level (Table 1). The means for these treatments were not significantly different (P > 0.05) than those for continuous aeration treatment for both irrigation levels.

### Root dry weight and activity

Table 2 shows the results of measurement of root dry weight at 58, 85, and 99 DAS. The 2-way ANOVA showed irrigation level treatments had no significant effect on root dry weight on any of these dates and there were no irrigation ×aeration interactions. The aeration treatment effects were significant for all three dates. The aeration treatment means in Table 2 represent a 1-way ANOVA on the root dry weight observations at a given date at each irrigation level. Although not significantly different the root dry weight means at the high irrigation level were greater than at the low irrigation level for all aeration treatments (Table 2). As would be expected dry root weight values consistently increased with age of the plants. Aeration treatment effects were more pronounced for observations at 58 and 99 DAS. Aeration application between from 58 to 85 DAS significantly enhanced root dry weight for both irrigation levels on these dates.

**Table 2:** Root dry weight (g per plant) of potted single tomato plants (n = 3) measured at 57, 85, and 99 days after sowing (DAS) for the 6 post-infiltration aeration treatments (i.e. none or 2.5 liter aeration applied during 5 different periods) at the low and high irrigation levels (i.e. subsurface drip-irrigation to maintain soil at 60 to 70% and 70 to 80% of volumetric field capacity).

Aeration period, days after sowing	Root dry weight (g per plant) measured at givenDAS					
irrigation level	Low			High		
DAS	57	85	99	57	85	99
None	2.89a	3.52b	3.94c	3.14a	3.81b	4.13b
27–33	3.30a	3.89ab	4.37abc	3.31a	4.23ab	4.77ab
34–57	4.07a	4.72ab	5.28ab	4.16a	4.98ab	5.85a
58–85	3.09a	4.98a	5.55a	3.22a	5.18a	6.30a
86–99	3.05a	3.68ab	4.02bc	3.21a	4.18ab	4.74ab
27–99	3.90a	4.74ab	5.22abc	4.22a	5.13a	5.81a
OVERALL MEAN	3.38	4.26	4.73	3.54	4.58	5.27

*Notes*: Irrigation and interaction effects were not significant (p>0.05) in ANOVA. Aeration treatment means at each irrigation level not followed by the same letter are significantly different at the 5% level

The 2-way ANOVA showed both irrigation level and soil aeration treatments significantly affected root activity observations at 58, 85, and 99 DAS. Also, the irrigation ×aeration effects were significant for these 3 dates (Table 3). Root activity was markedly higher at 85 DAS compared to observations at 58 and 99 DAS. At 57 DAS the root activity for all aeration treatment at the high irrigation were significantly greater than corresponding values for the low irrigation level (Table 3).

**Table 3:** Irrigation × post-infiltration aeration interaction on root activity (as mg triphenyl formazan per g fresh root per hour) of potted single tomato plants (n = 3) measured at 57, 85, and 99 days after sowing (DAS). Aeration treatments (i.e. none or 2.5 liter aeration applied during 5 different periods) at the low and high irrigation levels (i.e. subsurface drip-irrigation to maintain soil at 60 to 70% and 70 to 80% of volumetric field capacity).

Aeration period, days after sowing	Root activity (TPF per g fresh root per hour) for irrigation level at given DAS								
DAS	57			85			99		
irrigation level	Low	High	t-test	Low	High	t-test	Low	High	t-test
None	7.4 c	7.8 d	*	11.6 e	11.8 d	ns	6.7 c	6.9 c	ns
27–33	8.3 b	8.8 c	*	12.5 d	12.4 c	ns	6.8 c	6.7 c	ns
34–57	9.4 a	10.4 a	**	13.5 b	14.7 b	**	9.2 a	9.2 b	ns
58–85	7.4 c	9.3 b	**	12.9 c	15.1 a	**	9.4 a	9.6 a	ns
86–99	6.9 d	7.7 d	**	11.6 e	11.8 d	ns	6.7 c	6.8 c	ns
27–99	8.4 b	9.3 b	*	14.9 a	15.3 a	*	8.6 b	9.1 b	*
OVERALL MEAN	6.8	7.6	**	11.0	11.6	**	6.8	6.9	ns

*Notes*: Aeration treatment means at each irrigation level (n = 3) not followed by the same letter are significantly different at the 5% level. The t-test was used to compare 2 irrigation treatment means (n = 3) for each aeration treatment period. The asterisk indicates significantly different means (* for p ≤ 0.05) and (** for p ≤ 0.01, otherwise not significant (ns).

### Titratable acidity and vitamin C content

ANOVA results for titratable acidity and vitamin C levels showed highly significant irrigation ×aeration effects (p = 0.02 and p < 0.01 respectively). Post-infiltration aeration effects were highly significant (p ≤ 0.01) for both response variables, as was the irrigation level effect, but only for titratable acidity. The post-infiltration aeration effects at each irrigation level on these two fruit nutritional properties are presented in Table 4.

**Table 4:** Irrigation × post-infiltration aeration interaction on titratable acidity and vitamin C content (fresh weight basis) of the first ripe fruit from the first truss of potted single tomato plants (n = 3). Aeration treatments were none or 2.5 liter aeration applied during 5 different periods. Pots were subsurface drip-irrigated at 2-day intervals to maintain the soil at 60 to 70% or 70 to 80% of volumetric field capacity (denoted as low and high irrigation level).

Aeration period, days after sowing	Titratable Acidity(weight %)			Vitamin C Content(mg /100g)		
Irrigation level	Low	High	t-test	Low	High	t-test
None	0.79a	0.82a	ns	55.0a	42.8a	*
27–33	0.76b	0.78b	ns	69.4b	59.3b	*
34–57	0.62c	0.65d	ns	67.7b	59.2b	*
58–85	0.65c	0.73c	*	59.8a	71.5c	*
86–99	0.65c	0.64d	ns	57.8a	64.9b	*
27–99	0.62c	0.69c	*	60.5a	64.4b	ns
OVERALLMEAN	0.68	0.72	**	61.7	60.4	ns

*Notes*: Aeration treatment means at each irrigation level (n = 3) not followed by the same letter are significantly different at the 5% level. The t-test was used to compare 2 irrigation treatment means (n = 3) for each aeration treatment period and across aeration treatments (n = 18). The asterisk indicates significantly different irrigation means (* for p ≤ 0.05), (** for p ≤ 0.01, otherwise not significant (ns).

The overall mean (n = 18) for % titratable acidity was significantly higher (0.68% versus 0.72% with LSD_0.05_ = 0.02) at the high irrigation level. On the other hand there was no significant difference in the overall mean vitamin C content, which was slightly lower at the high irrigation level (61.7 versus 60.4 mg / 100 g with LSD_0.05_ = 2.5). As shown in Table 4, titratable acidity means (n = 3) were consistently greater at the high irrigation level for all aeration treatments but were significantly different (LSD_0.05_ = 0.04) only for aeration during fruit enlargement (58–85 DAS) and during the entire growth period (27–99 DAS).

The results (Table 4) further show that at both irrigation levels, titratable acidity was significantly higher for post-infiltration aeration during the earlier growth stages (27–33 DAS) and for the no aeration treatment relative to the later stages. For the low irrigation level, vitamin C content was also higher for the aeration treatment during the earlier growth stages (27–33 DAS and 34–57 DAS) than for the no aeration and aeration at the later stages. On the other hand, vitamin C content was maximum at the high irrigation level for aeration during the fruit enlargement stage (58–85 DAS).

### Shape and hardness

The ANOVA results for experimental treatment effects on the fruit axial and lateral diameters, and hardness (measured on the first 8 ripe fruits harvested from the first trusses of 3 potted single tomato plants) show no significant irrigation nor irrigation ×post-infitration aeration effect (Table 5). Although the irrigation treatment differences were non-significant, means (n = 3) for each aeration treatment were higher at the high irrigation level (Table 5). Overall mean axial diameter (n = 48) across aeration treatments was 44.6 mm and 46.4 mm for the low and high irrigation levels. Corresponding values for lateral diameter were 56.9 and 58.8 mm, and 3.6 and 3.7 N mm^-2^ for fruit hardness measured as penetrometer resistance.

**Table 5:** Axial and lateral diameter, and hardness measured on the first 8 ripe fruits harvested from the first trusses of 3 potted single tomato plants for 6 post-infiltration aeration treatments (i.e. none or 2.5 liter aeration applied during 5 different periods). Pots were subsurface drip-irrigated at 2-day intervals to maintain the soil at 60 to 70% or 70 to 80% of volumetric field capacity (denoted as low and high irrigation level).

Aeration period, days after sowing	Axial diameter,(mm)			Lateral diameter,(mm)			Hardness,(N mm^-2^)		
Irrigation level	Low	High	Mean (n = 16)	Low	High	Mean(n = 16)	Low	High	Mean(n = 16)
None	37.7a	38.4a	38.0a	53.2a	55.7a	54.5a	2.7a	2.9a	2.8a
27–33	44.0ab	45.5ab	44.7b	56.8a	58.2a	57.5a	3.6b	3.7ab	3.7b
34–57	48.4b	51.3b	49.9b	59.8a	60.6a	60.2a	3.8b	4.1b	3.9b
58–85	47.0ab	50.2b	48.6b	58.9a	62.2a	60.5a	4.0b	4.0b	4.0b
86–99	43.7ab	44.9ab	44.3b	54.9a	55.7a	55.3a	3.6b	3.6ab	3.6b
27–99	47.2ab	48.2b	47.7b	58.1a	60.1a	59.1a	3.9b	4.0ab	3.9b
OVERALLMEAN	44.6	46.4	45.5	56.9	58.8	57.9	3.6	3.7	3.6

*Notes*: Irrigation and interaction effects were not significant (p>0.05) in ANOVA. Aeration treatment means across irrigation levels (n = 16) not followed by the same letter are significantly different at the 5% level.

The axial diameter and hardness of fruits from the non-aerated plants was significantly lower (P ≤ 0.05) than those from their post-infiltration aerated counterparts. The mean axial diameter (n = 16) across the low and high irrigation levels for the non-aerated plant fruits was 38.0 mm and significantly less than the values for all the other post-infiltration aeration treatments. Post-infiltration aeration during 34–57 DAS resulted in a 31% increase in axial diameter, followed by increases of 28% and 26% for aeration during the 58–85 DAS and 27–99 DAS stages respectively (Table 5). Fruit hardness responded similarly to the post-infiltration aeration treatments. Aeration during the 34–57 DAS and 58–85 DAS stages gave mean values (n = 16) of 3.9 and 4.0 N mm^-2^. Both values were 43% higher than the hardness of 2.8 N mm^-2^ for the non-aerated plant fruits (Table 5). Aeration treatments had no significant effect on lateral diameter (Table 5). Nevertheless, the differences between the aeration treatment means across irrigation levels for lateral diameter (n = 16) showed a similar pattern as for the axial diameter and hardness (Table 5).

## Discussion

### Fresh fruit yield

The non-significant irrigation effect on fresh fruit yield indicated adequate soil water availability at both the low and high levels. Using results reported by Rawls et al.[34], the estimated volumetric water content at field capacity of silty clay loam soil in the pots = 0.50, and volumetric water content = 0.21 at soil water potential of -1.5 MPa (considered as the permanent wilting point). Given a total volume of 9.8 liter soil in the pots, these properties imply plant available water between 0.9 and 1.4 liter at the low irrigation level (i.e. 0.6 to 0.7 of field capacity), and between 1.4 and 1.9 liter at the high irrigation level (i.e. 0.7 to 0.8 of field capacity). Between 27–99 DAS, the mean ± standard deviation (n = 18) for the total volumes of water applied per pot = 36.9 ± 2.2 liter for the low irrigation and 39.3 ± 4.4 liter for the high irrigation. This implies an average irrigation rate of about 512 cm^3^ per pot per day for the low irrigation level and 546 cm^3^ per pot per day for the high irrigation level. Transpiration rates averaged 300 to 350 cm^3^ per day over the growing period for single potted tomato plants grown under similar conditions[35]. Hermanto et al.[36] reported similar rates of 0.3–0.4 liter plant^-1^ day^-1^ for greenhouse produced tomato under drip irrigation. Soil water stress was therefore unlikely for both the low and high irrigation levels during the growth period in this experiment, and would most likely explain the observed weak effect of irrigation levels on fresh fruit yield.

No previous study had specifically examined the response of sub-surface drip-irrigated potted tomato plants at different growth stages to post-infiltration soil aeration. Increasing levels of drip irrigation at a given frequency would supply more soil water available for transpiration, and generally this would increase growth and yield. On the other hand increasing drip-irrigation levels also result in lower levels post-infiltration soil aeration. Rawls et al.[34] reported a geometric mean bubbling pressure of 33 for 689 samples silty clay loam soils. Since there was 22.5 cm depth of soil in the pots, it would be expected that there would be little tendency for gravitational drainage from the pots, and that the soil in the vicinity of the sub-surface drip emitters would be close to saturation immediately after irrigation. Since redistribution in finer textured soils is generally slow, this saturated zone would be expected to persist for some time after irrigation. This extent of this zone would also increase with increasing irrigation volumes required to compensate for consumptive use by the plants over time, and maintain the target volumetric soil water content. Any positive effect of post-infiltration aeration would be related primarily on how well the injected air stream permeates this zone and promotes enhanced root activity and overall plant growth and development.

### Root dry weight and activity

The results on root dry weight and activity tend to support this conclusion. The taproot of tomato transplants are usually broken and this results in a fibrous-like system as a result of the an increased density of the laterals in the first few inches[37]. For transplants in the field, they observed close to 10 short laterals per cm averaged over the upper 15 cm of the taproot for tomato plants 4 weeks after transplanting in the field. These laterals branched profusely resulting in the surface soil in a 30 cm radius around the plant thoroughly ramified with rootlets. For subsurface drip irrigation in the field with the emitter directly beneath the plant row Hanson and May[38] found tomato plant roots were concentrated around the drip lines. Dresbøll Thorup-Kristensen[39] found that during short-term hypoxia in part of the root system, potted tomato plants showed increased activity in the non-affected roots to compensate for suboptimal conditions. Post-infiltration aeration would more likely ameliorate hypoxic conditions above, rather than below, the emitter. In this study, the root activity was measured on root tip samples taken from the entire root system. This most likely explains the significant and rather complicated interaction between post-infiltration aeration and irrigation level on root activity as the size of the root system increases over time in the confined volume of the pots. It also explains the consistent marked differences between the none and 27–99 DAS aeration treaments (Table 3).

These results also indicate that the sensitivity of the potted tomato plant roots may change as the roots system develops over time. Niu et al.[27] showed that soil aeration increases the oxygen content in the soil, and significantly enhance root activity and root absorption capacity. This research shows that post-infiltration soil aeration increased root activity especially during the fruit setting (34–57 DAS) and enlargement (58–85 DAS) growth stages. Aeration from 86 to 99 DAS almost no change of root activity, and root activity of aerated at the whole growth stage was lower than aerated at fruit setting (34–57 DAS) and enlargement (58–85 DAS) growth stages. This is fully shows the main stage of root needs oxygen is fruit setting (34–57 DAS) and enlargement (58–85 DAS) growth stages.

### Soluble solids

The overall soluble solids content (as °Brix) of the blended fruit across all treatments was 5.17 (Table 1) and comparable to the range in values (4 to 6 °Brix) reported for large round fresh market tomato varieties[40,41]. Water, soluble and insoluble solids, and organic acids are the main constituents of tomato fruits. Sweetness, sourness, and saltiness taste sensors on the human tongue respond to level and proportions of dissolved sugars, inorganic salts, and acids[42]. As a result, the soluble solids content and titratable acidity are the major nutritional parameters (termed as macro-components) controlling consumer preferences for fresh tomato fruit. The soluble solids are mostly free reducing sugars such as glucose and fructose and soluble polysaccharides. The insoluble constituents are insoluble polysaccharides (pectins, hemicelluloses, cellulose) that are linked and networked together to make up the cell wall and mechanical properties of the fruit. Citric and malic acids are the main organic acids along with minor contribution of tartaric, acetic, and oxalic acids. Vitamin C (as reduced ascorbic acid) is considered the main dietary micro-component[3,32]. The levels of these components have been shown to depend on cultivar, soil and environmental factors, and production practices such as fertilizer, irrigation, maturity at harvest, and post-harvest handling[32,40,43]. Despite these empirical findings, definitive biochemical and biophysical models to describe and predict such effects remain elusive, mainly because genetics control the plant physiological response.

These results add to the previous report suggesting that post-infiltration aeration beneficially impacted yield even when applied during different stages of the growing period. It appears that post-infiltration aeration can have positive effects on potted drip-irrigated tomato, not only on fresh fruit yield, but also on fruit nutritional quality. It is well-documented that root zone hypoxia and inadequate soil water availability rapidly induces stomatal closure[14,16,25,44,45]. Since stomatal closure would reduce air exchange in the leaves and reduce sugar synthesis, and this may explain the positives effect of post-infiltration aeration at the different growth stages on soluble solids content and on fresh fruit yield.

### Titratable acidity and vitamin C content

Most of the titratable acidity in tomato fruit consists of tri-carboxylic citric acid (C_6_H_6_O_7,_ 192 g mol^-1^) and di-carboxylic malic acid (C_4_H_6_O_5,_134 g mol^-1^). Reaction of citric acid with 0.1 M NaOH (4 g NaOH per liter) implies 1 ml NaOH equivalent to 0.0064 g citric acid. Similarly, 1 ml 01 M NaOH is equivalent to 0.0067 g malic acid. The weight % of titratable acidity in the blended fruit is therefore expressed as citric acid when calculated as (ml NaOH ×acid factor = 0.0064) divided by ml aliquot of blended fruit (assuming a density of 1 g cm^-3^ is for the blended fruit). The overall mean titratable acidity over all treatments in this experiment was 0.66% by weight with values ranging from 0.62 to 0.82 (Table 4). These values are comparable to those repored by Watada[46] and Paulson[47]. They reported values ranging from 20 to 56 mmol liter^-1^ citric acid and 3 to 12 mmol liter^-1^ malic acid in the ripe fruit juice of a large number of fresh market tomato cultivars. When combined, their values are equivalent to 22 to 64 mmol liter^-1^ titratable acidity expressed as citric acid or 0.42 to 1.23% by weight. In general, titratable acidity levels decreases with increasing ripeness and consumers mostly prefer lower levels.

The significant irrigation ×post-infiltration aeration effects obtained for titratable acidity and vitamin C content (Table 4) have not been previously reported. The vitamin C ranged from 43 to 72 mg per 100 g fresh fruit with overall mean value of 61. Studies have reported vitamin C content from 15 to 30 mg per 100 g for a range of fresh market tomato genotypes[46]. Genetically, vitamin C content (as well as soluble solids, type and level of titratable acidity, and other nutritional and taste parameters) in tomato is a quantitative trait[48]. The cultivar Tianze Chunlei used in this study appears to be genetically favored for vitamin C content. However, in common with other fruits and vegetables, climatic conditions, cultural practices etc. modify gene expression for vitamin C content[49].

Clearly, these results have to be somehow linked to physiological response to the spatial distribution of air and water in the root zone following irrigation and post-infiltration aeration application. One would expect that intensity of any such response would change over time as the root system expands logistically and occupies an increasing volume of soil. Studies have shown that management practices that affect water and air in the root zone can and does influence physiology of tomato plants(*Solanum lycopersicum*) [50,51]. These authors report that soil water deficits can improve fruit nutritional and taste quality (as indicated by soluble solids, organic acids, and vitamin C content) to varying extents. Nevertheless, further insights are needed about the spatio-temporal interplay of air and water in the root zone of the plants under the conditions of this experiment to fully explain the observed interaction between irrigation level and post-infiltration aeration at the different growth on fruit nutritional and taste parameters.

### Shape and hardness

As opposed to nutritional and taste, market quality is primarily visual. Consumers' decision when purchasing fresh tomato and other fruits and vegetables is influenced by factors such as size, shape, color, feel etc. Consumers generally prefer moderately sized, almost spherical, and firm-fleshed tomato fruits. The fruits of the tomato cultivar *Tianze Chunlei* used in this study has a circular lateral cross-section and is somewhat flattened axially such that the maximum lateral diameter is greater than the axial diameter (i.e. along the axis joining the stem and calyx ends of the fruit). Sphericity of such fruits increases as the ratio of these diameters becomes closer to equality. Consumers who prefer a spherical tomato would therefore select fruits with a ratio close to 1. The post-infiltration aeration treatments had no significant effect on lateral diameter but tended to significantly increase the axial diameter. This would indicate that post-infiltration aeration tended to increase the sphericity of the tomato fruits under the conditions of this experiment. A similar effect has not been reported elsewhere in the literature.

Several anatomical factors (such as cell wall structure, cuticle properties, turgor) determine tomato fruit hardness for a given cultivar[52,53]. Environmental factors are also involved, and their impacts are often hormonally mediated[54]. However, there are no reports in the literature that explain how root-zone hypoxia may impact tomato fruit hardness immediately after harvest. On the other hand it is well-known that inadequate root zone aeration induces hormonal signaling (such as cytokinins, gibberellins, auxins, ethylene, abscisic acid) that inhibits movement of substances (such as water, nutrients, and sugars) from root to shoot and vice-versa[55,56,57,58,59,60]. The experimental findings in this study suggest that such two-way biochemical root/shoot interactions may be involved.

In their entirety, these results show that post-infiltration aeration especially during the fruit setting and enlargement growth stage can positively influence the nutritional, taste, and market quality of tomato fruits produced in potted culture with sub-surface drip irrigation. Full explanation of interactions between irrigation level and post-infiltration aeration on some of these fruit quality parameters would require further insights about the dynamic interplay of air and water in the root zone on the plant physiology under the conditions of this experiment.

## Conclusions

Although an increasing number of studies have focused on the importance of rootzone O_2_ to plant growth and yield, few have reported on the effect of soil aeration on fruit nutritional and market quality. None have reported on post-infiltration aeration effects at different growth stages on these important aspects of tomato fruit quality. Our results showed that:

- Post-infiltration soil aeration (as opposed to none) applied during the entire growing period can positively impact tomato root dry weight and activity, and the fruit quality parameters. Aeration increased root dry weight and activity, and the fruit soluble solids, vitamin C content, axial diameter, and hardness, but decreased the titratable acidity content of sub-surface trickle-irrigated potted tomato.
- Post-infiltration aeration, applied during either the fruit setting (34–57 DAS) or enlargement (58–85 DAS) growth stages, can produce similar effects (as for the entire period) on the nutritional (soluble solids and vitamin C content), taste (titratable acidity), and market quality (shape and hardness) of tomato fruits produced under these conditions.

## Supporting Information

S1 Table Fruit yield, above-ground plant dry weight (g per plant), plant height (cm) and soluble solids content of the first ripe fruit from the first truss of potted single tomato plants for 6 post-infiltration aeration treatments (i.e. none or 2.5 liter aeration applied during 5 different periods) at 99 DAS. Pots were subsurface drip-irrigated at 2-day intervals to maintain the soil at 60 to 70% or 70 to 80% of volumetric field capacity (denoted as low and high irrigation level).
(XLSX)

S2 Table Root dry weight (g per plant) of potted single tomato plants (n = 3) measured at 57, 85, and 99 days after sowing (DAS) for the 6 post-infiltration aeration treatments (i.e. none or 2.5 liter aeration applied during 5 different periods) at the low and high irrigation levels (i.e. subsurface drip-irrigation to maintain soil at 60 to 70% and 70 to 80% of volumetric field capacity). (XLSX)

S3 Table Irrigation × post-infiltration aeration interaction on root activity (as mg triphenyl formazan per g fresh root per hour) of potted single tomato plants (n = 3) measured at 57, 85, and 99 days after sowing (DAS). Aeration treatments (i.e. none or 2.5 liter aeration applied during 5 different periods) at the low and high irrigation levels (i.e. subsurface drip-irrigation to maintain soil at 60 to 70% and 70 to 80% of volumetric field capacity).
(XLSX)

S4 Table Irrigation × post-infiltration aeration interaction on titratable acidity and vitamin C content (fresh weight basis) of the first ripe fruit from the first truss of potted single tomato plants (n = 3). Aeration treatments were none or 2.5 liter aeration applied during 5 different periods. Pots were subsurface drip-irrigated at 2-day intervals to maintain the soil at 60 to 70% or 70 to 80% of volumetric field capacity (denoted as low and high irrigation level).
(XLSX)

S5 Table Axial and lateral diameter, and hardness measured on the first 8 ripe fruits harvested from the first trusses of 3 potted single tomato plants for 6 post-infiltration aeration treatments (i.e. none or 2.5 liter aeration applied during 5 different periods). Pots were subsurface drip-irrigated at 2-day intervals to maintain the soil at 60 to 70% or 70 to 80% of volumetric field capacity (denoted as low and high irrigation level).
(XLSX)

## References

[1] Marrero TR, Mason EA. Gaseous diffusion coefficients. J Phys Chem A.1972;1(1):1–117.

[2] Cussler EL. Diffusion: Mass transfer in fluid systems 2nd ed. New York: Cambridge University Press;1997.

[3] Heuvelink E. Tomatoes Book 13 in the Crop Production Science in Horticulture series. Cambridge, Massachusetts, USA: CAB International Publishing;2005.

[4] Chang J, Wu X, Wang Y, Meyerson LA, Gu BJ, Min Y, et al Does growing vegetables in plastic greenhouses enhance regional ecosystem services beyond the food supply?. Front Ecol Environ.2013; 11(1):43–49.

[5] Maboko MM, Plooy CP. Effect of pruning on yield and quality of hydroponically grown cherry tomato (*Lycopersicon esculentum*).S Afr J Plant Soil.2008;25(3):178–181.

[6] Mbonihankuye C, Kusolwa P, Msogoya TJ. Assessment of the effect of pruning systems on plant developmental cycle, yield and quality of selected indeterminate tomato lines. Acta Hortic (ISHS).2013;1007:535–542.

[7] Bhattarai SP, Su N, Midmore DJ. Oxygation unlocks yield potentials of crops in oxygen-limited soil environments. Adv Agron.2005;88:313–377.

[8] Bryce JH, Focht DD, Stolzy LH. Soil aeration and plant growth response to urea peroxide fertilization. Soil Sci.1982;134 (3):111–116.

[9] Walter S, Heuberger H, Schitzler WH. Sensibility of different vegetables to oxygen deficiency and aeration with H_2_O_2_ in the rhizosphere. Acta Hortic.2004;659:499–508.

[10] Wang JF, Kang SZ, Li FS, Zhang FC, Li ZJ, Zhang JH. Effects of alternate partial root-zone irrigation on soil microorganism and maize growth. Plant Soil.2008;302:45–52.

[11] Wen GJ, Cai HJ, Chen XM, Liu HY. Influence of aeration irrigation on growth and fruit quality of greenhouse tomato. Journal of Northwest A & F University (Natural Science Edition).2013;41(4):113–118.(in Chinese with English abstract)

[12] Blom CWPM, Voesenek LACJ. Flooding: the survival strategies of plants. Trends Ecol Evol.1996; 11:290–295. 21237846 10.1016/0169-5347(96)10034-3

[13] Drew MC. Oxygen deficiency and root metabolism: injury and acclimation under hypoxia and anoxia. Annu Rev Plant Physiol Plant Mol Biol.1997; 48:223–250. 15012263 10.1146/annurev.arplant.48.1.223

[14] Sojka RE, Stolzy LH. Soil-oxygen effects on stomatal response. Soil Sci.1980;130(6):350–358.

[15] Bradford KJ, Yang SF. Physiological response of plants to waterlogging. HortScience. 1981;16:25–30.

[16] Bradford KJ, Hsiao TC. Stomatal behavior and water relations of waterlogged tomato plants. Plant Physiol.1982; 70:1508–1513. 16662706 10.1104/pp.70.5.1508 PMC1065914

[17] Kozlowski TT. Effects of flooding on growth and metabolism of herbaceous plants New York: Academic Press p. 47–128.

[18] Ashraf MA. Waterlogging stress in plants: A review. Afr J Agr Res.2012;7:1976–1981.

[19] Costa JM, Heuvelink E. The tomato crop and industry Massachusetts, USA: CAB International Publishing; 2005 p.1–19.

[20] Rao R, Li Y. Management of flooding effects on growth of vegetables and selected field crops. Horttechnology.2003;13(4):610–616.

[21] Karlen DC, Sojka RE, Robbins ML. Influence of excess soil water and N rates on leaf diffusive resistance and storage quality of tomato fruit. Commun Soil Sci Plan.1983;14(8):699–708.

[22] Ezin V, Pena RDL, Ahanchede A. Flooding tolerance of tomato genotypes during vegetative and reproductive stages. Braz J Plant Physiol.2010;22(2):131–142.

[23] Bhattarai SP, Salvaudon C, Midmore DJ. Oxygation of the Rockwool Substrate for Hydroponics. Aquaponics Jomnal.2008;49: 29–33.

[24] Bhattarai SP, Midmore DJ, Pendergast L. Water-use efficiencies and root distribution of soybean, chickpea and pumpkin under different subsurface drip irrigation depths and oxygation treatments in vertisols. Irrigation sci.2008;26:439–450.

[25] Bhattarai SP, Pendergast L, Midmore DJ. Root aeration improves yield and water use efficiency of tomato in heavy clay and saline soils. Sci Hortic.2006;108(3):278–288.

[26] Su NH, Midmore DJ. Two-phase flow of water and air during aerated subsurface drip irrigation. J Hydrol.2005;313:158–165.

[27] Niu WQ, Jia ZX, Zhang X, Shao HB. Effects of soil rhizosphere aeration on the root growth and water absorption of tomato.Clean-Soil, Air, Water.2012;40(12):1364–1371.

[28] Meek BD, Ehlig CF, Stolzy LH, Graham LE. Furrow and trickle irrigation: Effects on soil oxygen and ethylene and tomato yield. Soil Sci Soc Am J.1983;47(4):631–635.

[29] Chen JP, Liu ZG, Duan AW, Meng ZJ, Zhang JY. Effects of soil moisture on physiological characteristics and the dynamic state of factors causing photosynthesis decline in potted tomato leaves in green house. Acta Botanica Boreali-occidentalia Sinica.2004;24(9):1589–1593.(in Chinese with English abstract)

[30] Zhao SJ, Liu HS, Dong XC. Guidance of Physical Experiment of Plants.Beijing: China Agricultural Science and Technology Press 1998; p.56–124. (in Chinese)

[31] Pieper JR, Barrett DM. Effects of organic and conventional production systems on quality and nutritional parameters of processing tomatoes. J Sci Food Agr.2009;89:177–194.

[32] Gould WA. Tomato production, processing, and technology 3rd ed. Baltimore, USA:CTI Publications;1992.

[33] Gao JF. Experimental Guidance for plant physiology Beijing: Higher Education Press; 2006 p.203–204. (in Chinese)

[34] Rawls WJ, Brakensiek DL, Saxton KE. Estimation of soil water properties.Transactions Amer Soc Agricultural Engineers.1982;25(5):1316–1320.

[35] Nahar K, Ullah SM. Morphological and physiological characters of tomato (*Lycopersicon esculentum Mill*.) cultivars under water stress. Bangladesh J Agric Res.2012;37(2):355–360.

[36] Harmanto, Salokhe V, Babel M, Tantauc H. Water requirement of drip irrigated tomatoes grown in greenhouse in tropical environment. Agr Water Manage.2005;71:225–242.

[37] Weaver E, Bruner WE. Muskmelon In: Root development of vegetable crops. New York: Mcgraw-Hill Book Company 1927; p.180–196.

[38] Hanson B, May D. The effect of drip line placement on yield and quality of drip-irrigated processing tomatoes. Irrig Drainage Syst.2007;21(2):109–118.

[39] Dresbøll D, Thorup-Kristensen K. Spatial variation in root system activity of tomato (*Solanum lycopersicum L*.) in response to short and long-term waterlogging as determined by N uptake. Plant Soil.2012;357:161–172.

[40] Davis JN, Hobson GE. The constituents of tomato fruit-influence of environment, nutrition, and genotype. Crit Rev Food Sci.1981;14(5):205–281. 10.1080/10408398109527317 7030623

[41] Aldrich HT, Salandanan K, Kendall P, Bunning M, Stonaker F, Kulen O, et al Cultivar choice provides options for local production of organic and conventionally produced tomatoes with higher quality and anti-oxidant content. J Sci Food Agr.2010;90:2548–2555. 20718027 10.1002/jsfa.4116

[42] Hui YH editor. Handbook of fruit and vegetable flavors Hoboken, New Jersey: John Wiley & Sons;2010.

[43] Jongen W editor. Fruit and vegetable processing Abington, Cambridge, UK: Woodleaf Publishing Ltd.;2002.

[44] Bradford KJ. Effects of soil flooding on leaf gas exchange of tomato plants. Plant Physiol.1983;73:475–479. 16663242 10.1104/pp.73.2.475 PMC1066487

[45] Sojka RE. Stomatal closure in oxygen-stressed plants. Soil Sci.1992;154(4):269–280.

[46] Watada AE, Aulenbach BB, Worthington JT.Vitamins A and C in ripe tomatoes as affected by stage of ripeness at harvest and by supplementary ethylene. J Food Sci.1976;41:856–858.

[47] Paulson KN, Stevens MA. Relationships among titratable acidity, pH and buffer composition of tomato fruits. J Food Sci.1974;39: 354–357.

[48] Narolia RK, Reddy RVSK, Sujatha M. Genetic architecture of yield and quality in tomato (*Solanum lycopersicum*).Agr Sci Dig.2012;32(4):281–285.

[49] Seung KL, Kader AA. Preharvest and postharvest factors influencing vitamin C content of horticultural crops. Postharvest Biol Tec.2000;20:207–220.

[50] Shao GC, Wang MH, Liu N, Yuan M, Kumar P, She DL. Growth and comprehensive quality index of tomato under rain shelters in response to different irrigation and drainage treatments. The Scientific World J.2014;1–12. 10.1155/2014/457937 PMC4098618 25054180

[51] Chen JL, Kang SZ, Du TS, Qiu RJ, Guo P, Chen RQ. Quantitative response of greenhouse tomato yield and quality to water deficit at different growth stages. Agr Water Manage. 2013;130: 152–162.

[52] Hall CB. Firmness and color of some tomato varieties during ripening and according to harvest dates. Proc Amer Soc Hort Sci.1964;84:507–512.

[53] Ahrens MJ, Huber DJ. Physiology and firmness determination of ripening tomato fruit. Physiol Plantarum.1990;78(1):8–14.

[54] Chen JL, Kang SZ, Du TS, Guo P, Qiu RJ, Chen RQ, et al Modeling relations of tomato yield and fruit quality with water deficit at different growth stages under greenhouse condition. Agr Water Manage.2014;146: 131–148.

[55] Jackson MB.Root-to-shoot communication in flooded plants: involvement of abscisic acid, ethylene, and 1-aminocyclopropane-1-carboxylic acid. Agron J.1994;86(5):775–782.

[56] Morard P, Lacoste L, Silvestre J. Effect of oxygen deficiency on uptake of water and mineral nutrients by tomato plants in soilless culture. J Plant Nutr.2000;23(8):1063–1078.

[57] Horchani F, Khayati H, Raymond P, Brouquisse R, Aschi-Smiti S.Contrasted effects of prolonged root hypoxia on tomato root and fruit (*Solanum lycopersicum*) metabolism. J Agron Crop Sci.2009;195(4):313–318.

[58] Horchani F, Aloui A, Brouquisse R, Aschi-Smiti S.Physiological responses of tomato plants (*Solanum lycopersicum*) as affected by root hypoxia. J Agron Crop Sci.2008;194(4):297–303.

[59] Kläring HP, Zude M. Sensing of tomato plant response to hypoxia in the root environment. Sci Hort.2009;122 (1):17–25.

[60] Horchani F, Aschi-Smiti S. Prolonged root hypoxia effects on ethylene biosynthesis and perception in tomato fruit. Plant Signal Behav.2011;6(1):1–4. 21307662 10.4161/psb.6.1.13880 PMC3121994

